# Tinnitus Perception and Distress Is Related to Abnormal Spontaneous Brain Activity as Measured by Magnetoencephalography

**DOI:** 10.1371/journal.pmed.0020153

**Published:** 2005-06-28

**Authors:** Nathan Weisz, Stephan Moratti, Marcus Meinzer, Katalin Dohrmann, Thomas Elbert

**Affiliations:** **1**Department of PsychologyUniversity of KonstanzGermany; Tinnitus and Hyperacusis CentreUnited Kingdom

## Abstract

**Background:**

The neurophysiological mechanisms underlying tinnitus perception are not well understood. Surprisingly, there have been no group studies comparing abnormalities in ongoing, spontaneous neuronal activity in individuals with and without tinnitus perception.

**Methods and Findings:**

Here, we show that the spontaneous neuronal activity of a group of individuals with tinnitus (*n* = 17) is characterised by a marked reduction in alpha (8–12 Hz) power together with an enhancement in delta (1.5–4 Hz) as compared to a normal hearing control group (*n* = 16). This pattern was especially pronounced for temporal regions. Moreover, correlations with tinnitus-related distress revealed strong associations with this abnormal spontaneous activity pattern, particularly in right temporal and left frontal areas. Overall, effects were stronger for the alpha than for the delta frequency band. A data stream of 5 min, recorded with a whole-head neuromagnetometer under a resting condition, was sufficient to extract the marked differences.

**Conclusions:**

Despite some limitations, there are arguments that the regional pattern of abnormal spontaneous activity we found could reflect a tinnitus-related cortical network. This finding, which suggests that a neurofeedback approach could reduce the adverse effects of this disturbing condition, could have important implications for the treatment of tinnitus.

## Abstract 2

Regional patterns of abnormal brain activity identified in people with tinnitus suggest the presence of a tinnitus- related cortical network that it may be possible to modify by neurofeedback.

## Introduction

Tinnitus is characterised by the perception of sounds (e.g., a tone, hissing, or roaring noise, and sometimes combinations of such perceptions) in the absence of any objective physical sound source. For the affected individual, this condition often causes a considerable amount of distress. Although it is now widely accepted that the generation of tinnitus has a central basis [1–3], the neurophysiological mechanism is still not well understood. Typically, tinnitus is accompanied by an audiometrically measurable hearing loss, and the considerable overlap between hearing loss and tinnitus spectra [4] suggests that these phenomena may be related. Thus, a widespread assumption is that neuronal response properties in the auditory system change following damage to it. These changes may persist even after recovery from the peripheral lesion. However, there is no general agreement as to which changes are responsible for the perception of this auditory phantom phenomenon. Studies in humans and animals indicate that neurons in the deprived regions of the auditory cortex change both their receptive field [5–8] and their spontaneous activity [1,9,10]. Furthermore, recent positron emission tomography studies in particular have pointed to the brain areas involved in attentional and emotional regulation [11–14]. An example of such a neuroimaging study was conducted by Lockwood et al. [14], who investigated individuals with tinnitus who were able to enhance or reduce the perceived loudness of their tinnitus via oral–facial movements. Besides changes in auditory cortical activity contralateral to the affected ear, the authors reported changes in hippocampal activity related to loudness changes. The authors interpreted this as evidence for limbic system involvement. In addition to this study implicating the limbic system (and temporal) structures, studies by Mirz et al. [11,12] indicate that frontal areas have a role in tinnitus.

Surprisingly, even though spontaneous activity has been a frequent research target in animal models of tinnitus, studies in humans have been rare. Data from a group study for abnormal activity of the cochlear nerve were reported by Martin [15]. An abnormal peak in spectral power could be observed close to 200 Hz in a majority of participants undergoing cerebellopontine angle surgery, a majority of whom had tinnitus (in the present study, however, we mean cortical activity when speaking of spontaneous activity). When extracted from spontaneous magnetoencephalography (MEG)–produced images in perisylvian regions, the values of the largest Lyapunov exponent—a measure for the predictability of the time series—were generally larger for the participants with tinnitus than for control individuals, indicating different nonlinear temporal dynamics in spontaneous activity for tinnitus and control participants [16]. A further study aiming to use information offered by spontaneous ongoing brain rhythms was published by Shulman and Goldstein, who reported temporal and frontotemporal changes (increases and decreases) of relative power and coherence irregularities in severely disabled individuals with tinnitus [17]. Otherwise, attempts to study tinnitus in humans have focussed on the use of designs that measure neurophysiologic responses following sounds [8,18–23] or experimental manipulations that enhance or reduce the perceived loudness [13,14,24,25]. In the present study, we examined the power spectrum of neuromagnetic oscillatory activity during rest.

The generation of tinnitus, in most cases, can be linked to damage to the auditory system, usually to receptors of the inner ear [1–3], probably even in cases where an impairment cannot be assessed audiometrically [26]. In this sense, one can speak of the presence of a deafferentation in the system since certain areas of the brain are now deprived from their normal input. Various sources of evidence indicate that deprivation of primary input leads to the functioning of the system in a slow-wave modus, i.e., analysis of neuroelectric signals in the frequency domain reflect an enhanced power in the delta frequency range (< 4 Hz). A dramatic example of this fact is the presence of neurological damage, e.g., following an infarct or a tumor. In these cases, enhanced slow-wave activity can be detected in perilesional areas [27–31]. Another example is slow-wave sleep [32,33], during which thalamic (and thus also cortical) centers are cut off from input from prethalamic relays. This condition leads to a hyperpolarisation of thalamocortical cells [34] that activates sodium and potassium currents, gradually depolarising the cell. The depolarisation in turn triggers a calcium-mediated low-threshold spike burst. The frequency of this hyperpolarisation–spike-burst cycle is approximately in the delta to theta frequency range. Interestingly, hyperpolarisation-dependent low-threshold spike bursts in the thalamus have previously been associated with positive symptoms in various pathologic conditions including tinnitus [35,36]. Because of the corticothalamic connections, coherent slow-wave oscillations are also seen on a cortical level [36]. In the case of tinnitus, one would expect to find them within the temporal lobe. In the electroencephalogram, much of this activity would project to frontal sites because of the orientation of the sources in the auditory cortex, where it gets blurred as activity from other regions, especially radially oriented frontal sources, also contribute to and even dominate the electroencephalogram at this site. MEG recordings can be used to differentiate between radial frontal and tangential temporal sources, as MEG is largely blind to the former but not to the latter sources. Studies from slow-wave sleep also show that an enhancement of oscillatory activity in the delta band is accompanied by a reduced activity in several other frequency bands—among others, alpha, sigma, and beta [32,37]. Next to enhanced delta activity, attenuated alpha activity has been implicated in schizophrenia [38,39]. These results indicate that a change in the delta range might not be isolated phenomenon, but might instead be accompanied by reciprocal alterations in other frequency bands.

The data recorded by MEG represent the summed activity of tangential sources on a two-dimensional (sensor space) level. By applying various inverse solution strategies [40], it is possible to obtain information about potential (usually cortical) generators underlying the observed data. Using power spectrum analysis of such source-space-transformed neuromagnetic data the primary aim of the present study was to identify cortical regions with changes in underlying spontaneous activity patterns in individuals with tinnitus. Based on previous studies—summarised above—we predicted enhanced power in the delta frequency range. A second aim was to explore whether such an enhancement would also occur with a corresponding change in a different frequency band. Relations between the neurophysiologic scores and the subjective reports of tinnitus-related distress were examined by means of correlation analyses.

## Methods

### Participants

Seventeen individuals with chronic tinnitus (one woman; mean age ± standard error, 52.41 ± 2.70)—16 with high-frequency hearing loss and one with deafness due to transection of the hearing nerve—and 16 normal hearing control individuals (one woman; age 45.88 ± 3.84) participated in the study. Five minutes of resting MEG were measured and analysed with regard to the spectral content (see “Data Acquisition and and Signal Analysis” in Supporting Information for details). Tinnitus was reported to be bilaterally equal in four individuals, bilateral but left-dominant in one individual, and right-dominant in two individuals. Ten individuals reported unilateral tinnitus, of which eight indicated that they heard their tinnitus on the left side. The majority of individuals for which etiology is known experienced either a sudden hearing loss or noise trauma (assessed using a structured tinnitus interview). A comprehensive overview of all tinnitus participants is given in Table 1. Prior to the experiment, participants gave written informed consent. Tinnitus-related distress was assessed with a standard German questionnaire (Tinnitus Fragebogen [41], an adaption of the commonly used Tinnitus Questionnaire [42]), which allows tinnitus distress to be ranked on the following subscales: emotional distress, cognitive distress, intrusiveness, hearing problems, sleeping problems, and somatic complaints. In addition to the total score (sum of scores on each subscale), the following subscales were tested against the neurophysiological data separately: emotional distress, cognitive distress, intrusiveness, and sleeping problems. The last subscale, together with the regional slow-wave distribution, should help to rule out the hypothesis that changes in frequency bands could be a trivial result of sleep disturbances. Based on the total score, participants were assigned to a certain distress category: slight (0–30 points), moderate (31–46), severe (47–59), and very severe (60–84) distress. The greatest contributions (approximately 47%) to the total distress score (mean score ± standard error, 30.0 ± 4.69) were from emotional (7.8 ± 1.4) and cognitive distress (6.1 ± 0.9) and intrusiveness (approximately 27%; 8.0 ± 1.0)—i.e., factors that involve the perception and psychological effects of tinnitus more directly. Thus, 75% of the total score can be attributed to these three scales. Only 15% of the total distress score was attributed to reported hearing problems.

**Table 1:**
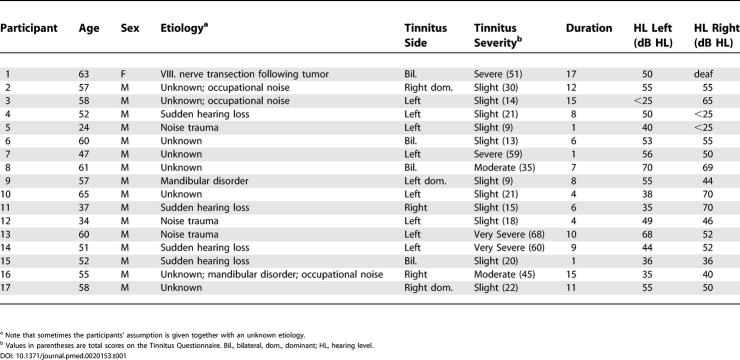
Participant Information

^a^ Note that sometimes the participants' assumption is given together with an unknown etiology.

^b^ Values in parentheses are total scores on the Tinnitus Questionnaire. Bil., bilateral, dom., dominant; HL, hearing level. DOI: 10.1371/journal.pmed.0020153.t001

### Statistics

We employed repeated-measures analysis of variance (assuming compound symmetry; this assumption was validated and superior to a general correlation structure) to test for the presence of main and interaction effects (group × frequency band) in the overall average for each frequency. Since the result of this analysis was significant, we executed this analysis for specified regions (see above) separately. Associations with tinnitus-related distress were tested using product-moment correlations. In a first step, the average over all dipoles for each frequency band was correlated with the tinnitus questionnaire. As this analysis yielded significant associations that were specific and consistent in direction for the alpha and delta band, values from each region were correlated with the scales of the questionnaire in order to help interpret effects gained in the first step.

## Results

### Spontaneous Neuronal Activity Pattern

**Figure 1:**
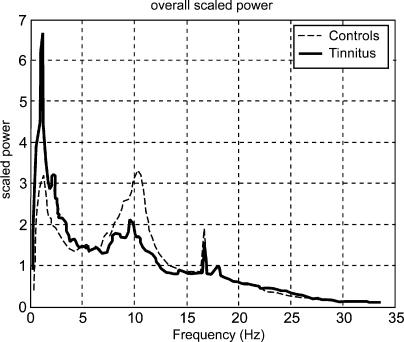
Power Spectra Averaged over All Sensors Show a Reduced Alpha Peak in Participants with Tinnitus and an Enhancement for Delta The sharp peak centred at 16 2/3 Hz represents technical noise resulting from the 1-km-distant railway system.

**Figure 2:**
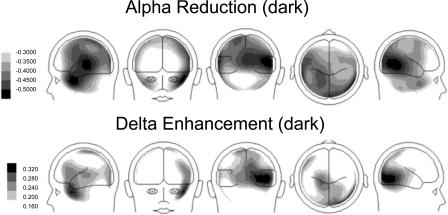
Difference Maps between Participants with Tinnitus and Controls for Alpha and Delta The results suggest that areas for which alpha reduction and delta enhancements are found partly overlap. Overall, the effect for the alpha band is considerably stronger (note that for this reason the scaling ranges are chosen differently).

**Figure 3:**
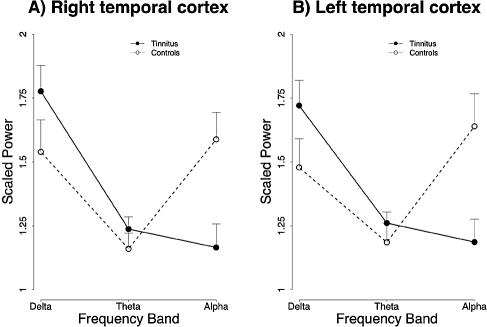
Display of the Group × Frequency Band Interaction Effects Averaged over Temporal Sources Effects for right (A) and left (B) temporal cortex, where the strongest enhancements of alpha and reductions of were found.

The explorative analysis of the frequency spectrum of the recorded magnetic fields revealed characteristic changes in participants with tinnitus from the usual pattern seen in normal hearing controls (Figure 1). The alpha peak, which can be seen in the control group, is strongly reduced in the tinnitus group. In contrast, the power in the delta frequency band is considerably enhanced. This pattern is spatially resolved by the results obtained by the minimum norm solution, which attributes both—the alpha attenuation and the delta enhancement—mostly to the temporal regions (Figure 2). It is clearly visible that the alpha reduction is considerably stronger than the delta enhancement. A repeated-measures analysis of variance reveals a significant group by frequency band interaction (*F*_2,62_ = 4.47, *p* < 0.02, power 0.78). Differences between the values of each frequency range (alpha versus theta, alpha versus delta, and theta versus delta) were calculated for each individual, and the results were subjected to a between-subjects analysis of variance. This analysis shows that the control group exhibited a significantly higher alpha than theta power (*F*_1,31_ = 5.84, *p* < 0.03), whereas the tinnitus group has stronger delta activity than alpha activity (*F*_1,31_ = 5.11, *p* < 0.03). No differences were found between the groups when theta was compared with delta (*F*_1,31_ < 1). These results statistically confirm the impression of a reduced alpha and enhanced delta peak in the tinnitus group. This interaction pattern was statistically significant for all regions, with the exception of right frontal and anterior parietal regions of both hemispheres. The foci of the altered spontaneous activity pattern in the tinnitus group are clearly found bilaterally in temporal areas (*F*_1,31_ > 5, *p* < 0.01), which can be seen in Figures 2 and 3.

### Association of Spontaneous Activity with Tinnitus-Related Distress

**Figure 4:**
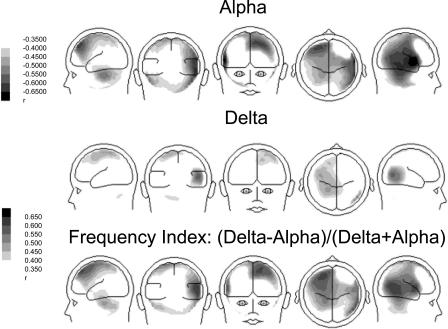
Correlation Map between Alpha, Delta, and Tinnitus-Related Distress Since previous analyses (see Figure 3) implicated corresponding areas for the effects found for alpha and delta, tinnitus-related distress was additionally correlated with a frequency index ([delta – alpha]/[delta + alpha]; bottom panel). Effects are largest for right temporal and left frontal sources.

For both alpha and delta power, significant correlations were found with the Tinnitus Questionnaire (total score and subscales), with *r* ranging between 0.5 and 0.7 (Table 2). These correlations correspond to large effect sizes, as outlined by Cohen [43]. Note, however, that because of the large number of calculations and the increased risk of false positives, *p*-values help us to gain a more general impression of the present associations. The distribution of correlation coefficients for the total score on the Tinnitus Questionnaire versus alpha and delta power is shown in Figure 4. Overall, correlations for alpha appear slightly stronger than for delta. Yet the strongest effects are located in similar brain regions (note that correlations smaller than 0.4 are coloured white). To exclude the possibility that the high associations are a consequence of potentially different subgroups—i.e., one subgroup high in delta and another one with low alpha—a frequency index was calculated ([delta – alpha]/[delta + alpha]) for each individual. The correlation of this score with the Tinnitus Questionnaire is displayed in the bottom panel of Figure 4. Strongest associations with this measure are found in right temporal and left frontal regions. Results of the theta band did not correlate with any of the questionnaire data. Also, correlations between hearing-loss parameters (depth and slope of hearing loss) and tinnitus-related distress were not significant (*p* > 0.4 for all). Amount and slope of hearing loss were also not significantly correlated with neuromagnetic data (0.12 < *r* < 0.25).

**Table 2:**
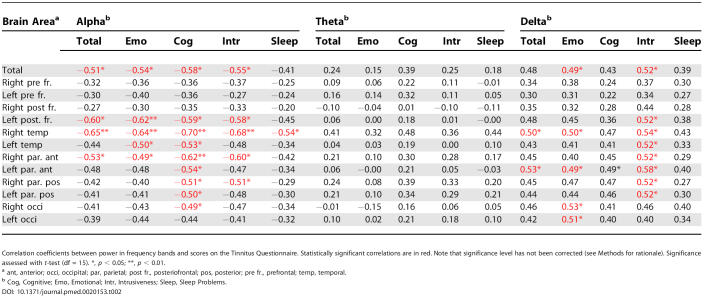
Correlations of Power with Tinnitus Questionnaire

Correlation coefficients between power in frequency bands and scores on the Tinnitus Questionnaire. Statistically significant correlations are in red. Note that significance level has not been corrected (see Methods for rationale). Significance assessed with *t*-test (df = 15). *, *p* < 0.05; **, *p* < 0.01.

^a^ ant, anterior; occi, occipital; par, parietal; post fr., posteriofrontal; pos, posterior; pre fr., prefrontal; temp, temporal.

^b^ Cog, Cognitive; Emo, Emotional; Intr, Intrusiveness; Sleep, Sleep Problems.

## Discussion

To our knowledge, this is the first group study on tinnitus in humans (there have been reports on single cases [35,36]) to show marked relative enhancements in delta and concomitant reductions in alpha spontaneous cortical activity. Notable group differences are a bilateral relative enhancement in delta and an accompanying reduction in alpha power over temporal areas (extending into posterior regions in the right hemisphere) in individuals with tinnitus. These findings are in agreement with a study conducted by Shulman and Goldstein [17], who report abnormalities in spontaneous activity in all 21 investigated patients. The definition of abnormality, however, was rather broad, meaning enhancements, reductions, or coherence irregularities in different bands. Another difference from our study is that these authors investigated severely disabled patients, whereas only a minority (four out of 17; see Table 1) of our participants could be classified in a similar manner. Our results are in agreement with ideas put forward by Jeanmonod and Llinas and colleagues [35,36], who stated that positive symptoms are a consequence of a hyperpolarisation of thalamic neurons following deafferentation, leading to spike bursts around 4 Hz. A look at the power spectra in Figure 1 shows that a reduced alpha cannot be caused by normalisation, because in this case the peak is almost absent—a pattern we encounter frequently in tinnitus participants. Concerning delta, it is very unlikely that the enhancement relative to alpha is an effect of the reduced alpha, as enhancements should then not be restricted to slow oscillations but should also be seen in other frequency bands (e.g., beta; note, e.g., that the artefactual 16-Hz activation is identical for both groups). Apart from this, the general pattern (i.e., alpha reduction and delta enhancement) is also seen in the non-normalised data (available upon request). It is unclear whether this relative enhancement of delta activity compared to alpha is the “abnormal activity” that is perceived as tinnitus [44]. The underlying neural substrate of tinnitus perception could also be an edge effect, such as gamma activity that arises in regions between abnormal (delta) and normal awake activity [45]. The (methodological) problem in the latter case lies, however, in the exact definition of gamma activity. However, it would be interesting to investigate the effects of treatments presumably inducing tinnitus on slow waves in animals. The fact that regions with an increase in slow-wave activity are also the regions of decreased alpha activity resembles results found during slow-wave sleep [32] and supports the idea that the changes in spontaneous brain activity might be mediated by sensory deprivation, i.e., partial hearing loss in this case. Additional evidence is provided by the observation that delta enhancement and alpha reduction were strongly correlated with tinnitus-related distress variables with a focus on the right temporal and also left frontal cortex. The association of the abnormality index—which sets alpha and delta power in relationship to one another—and tinnitus-related distress demonstrates that the effects reported are not an epiphenomenon of normalisation. Furthermore, a deviant abnormality index can be seen at an almost individual level. We are thus confident that the main effect reported here is not due to methodological flaws concerning data analysis.

However, we can not exclude with certainty the possibility that the effects are not specific to tinnitus, a limitation of our study. One interesting finding is that compared to the normal hearing control group, the tinnitus group also had a high-frequency hearing loss. So, theoretically, the ideal control group would have exactly the same type of hearing loss without a tinnitus sensation. However, to find such a group of an appropriate sample size would be very difficult as both phenomena are strongly associated. For example, in an attempt to investigate the influence of high-frequency hearing loss (similar to that of our study) on cortical reorganisation, Dietrich et al. [8] noticed that all hearing-impaired participants also had tinnitus. Yet it cannot be disputed that occasionally individuals exist who have a similar kind of deficiency in clinical audiograms as the participants in our study, but without having a tinnitus sensation. Simply having a reduced threshold does not seem to be sufficient to trigger reorganisational processes leading to tinnitus. Why these individuals (although rare) do not develop this symptom is a very important question for understanding tinnitus. A possible way to tackle this question is to analyse underlying hearing damage in more detail (e.g., amount of inner and outer hair-cell damage) by comparing individuals with hearing loss with and without tinnitus. Another strategy would be to investigate possible predisposing factors, e.g., psychological or genetic factors. As parameters of hearing loss (depth and slope) appear to be uncorrelated with distress scores and neurophysiological data, we assume its distorting effects to be minimal. This hypothesis is further supported by the fact that only a small proportion of the distress score was attributed to hearing loss. Overall, it should be emphasised that the issue of hearing loss is less serious in our study than in studies in which individuals with tinnitus (either human or animals) are acoustically stimulated (especially in the hearing-loss range).

The association of our neurophysiologic data with distress suggests that the right temporal and left frontal cortex might be involved in a tinnitus-related cortical network, in which the temporal region is associated more with perceptual issues (i.e., aspects concerning the character of the sound, e.g., tonal or noise-like, loudness), and the left frontal region more with affective distress and motivational attention of tinnitus (i.e., the tinnitus becoming a signal of high importance, so that it draws attention of the individual). Without reference to lateralisation, Jastreboff describes the prefrontal cortex as a “candidate for the integration of sensory and emotional aspects of tinnitus” [44]. Our data lend support to this notion. Concerning the stronger effect for the right temporal area than for the left, one has to consider the higher frequency of left-sided tinnitus in our study. Thus, it cannot be excluded that this asymmetry would vanish if more individuals with right-sided tinnitus were included in the analysis. However, the fact that tinnitus is generally more common for the left ear [46] certainly opens up possibilities to speculate about potential asymmetries, either on a peripheral or central level, that could account for this finding. The underlying physiological reasons for this asymmetry on an epidemiological level have not been a matter of research so far. Left frontal activation has been linked with positive, and right frontal activation with negative, affect [47–49]. In the context of this framework, enhanced alpha (indicating hypoactivation) in the left frontal cortex should be indicative of depression. Our alpha–distress association, however, is negative, thus implying that the results obtained cannot be explained by an effect of higher depressive mood in individuals with tinnitus. This simple explanation would also not fit with recent data from our group [52], which demonstrated a negative association between left frontal delta (measured via dipole density) and depression.

In future studies, we hope to elucidate the function of the (left) frontal area, as it may point us to the role of top-down influences that presumably play a role in the perception and perhaps even generation of tinnitus. An important aspect of these results is that they potentially have clinical implications, which are currently being tested by our group. Reducing the abnormal spontaneous activity pattern reported in this study via neurofeedback might cause concomitant reductions in tinnitus distress and/or intensity (often reported to be unrelated; [51]). Besides experimentally demonstrating to what extent our results reflect a “genuine” signature of tinnitus sensation in ongoing brain activity, these investigations could ultimately be of benefit for persons affected by this condition.

## Supporting Information

### Data Acquisition and Signal Analysis

Five minutes of MEG under a resting condition was recorded (sampling rate: 678.17 Hz; 0.1–200 Hz band-pass) using a 148-channel neuromagnetometer (Magnes 2500 WH, Biomagnetic Technologies, San Diego, California, United States). The participant was requested to keep eyes open and to maintain gaze on a fixation mark at the ceiling of the recording chamber. Eye movements were monitored with four electrodes attached to the left and right outer canthi and above and below the right eye.

In the first step of data analysis, sampling points were reduced by a factor of ten. After noise reduction, eye-movement correction was undertaken using the algorithm proposed by Berg and Scherg [52]. As an explorative step, spectral power was calculated for each sensor via mean fast Fourier transformations (window length, 7.55 s; 50% overlap between cosine squared windows). This step was taken to focus on specific frequency bands of interest. As the emphasis of the study was on alterations of spontaneous activity patterns within the tinnitus group, data were scaled by dividing each value by the overall mean power (gained by averaging over all sensors). As can be seen in Figure 1, participants with tinnitus showed a markedly reduced alpha peak accompanied by an enhancement in the slow frequency (delta) range.

The second step consisted of the investigation of the underlying source activity via application of the minimum norm estimate [53,54] to the eye-movement-corrected continuous data. This linear estimation technique yields a solution for the current density of a configuration of dipoles (here: 197 evenly distributed) located on a spherical shell by multiplying the pseudo-inverse of the lead-field matrix (which describes the sensitivity of each sensor to the sources) with the obtained data. The lead-field matrix was computed for each participant, based on information about the centre of a fitted sphere to the digitised head shape, and the positions of the MEG sensors relative to the head. In order to be able to average the obtained minimum norm estimate solutions over different participants, we employed a shell with a fixed radius of 6 cm. The radius of 6 cm was chosen as a tradeoff between depth sensitivity and spatial resolution [53]. Each of the 197 dipole locations consisted of two perpendicular dipoles oriented tangentially to the shell surface. The source-space transformed continuous data were then entered into the same mean fast Fourier transformation algorithm as described above. For both orientations of each dipole, power was calculated in the following frequency bands: delta (1.5–4 Hz), theta (4–8 Hz), and alpha (8–12 Hz). In order to gain a single value for each dipole, the square root of the sum of squares of the power for the two orientations was calculated and scaled in the same manner as described above (using mean power of each dipole instead of each sensor). Oscillatory activity was then analysed (a) averaged over all 197 dipoles and (b) in specific regions by grouping clusters of dipoles on the sphere and averaging their values. These regions (always bilateral) were prefrontal, frontal, temporal, frontocentral, parietal (anterior and posterior), and occipital cortex.

**Patient Summary**

**Background**

Tinnitus—hearing sounds such as hissing or roaring that are not really present—is a common condition and can be very distressing. Little is know about why tinnitus starts. Though people who have it often have some hearing loss, the hearing loss does not cause the tinnitus. There are few good treatments for tinnitus.

**What Did the Authors Do?**

They measured spontaneous activity in the brains of 17 people with tinnitus and 16 without. The found that one type of brain activity—alpha—was decreased and another—delta—was increased in people with tinnitus, particularly in one part of the brain, the temporal region, where the brain processes sounds. The changes were particularly pronounced in people who were most distressed by the tinnitus.

**What Do These Findings Mean?**

It seems that tinnitus is associated with one type of abnormal spontaneous activity in the brain. One way of treating tinnitus therefore might be to try to alter the activity by providing feedback about the ongoing brain activity and training the individual to interfere with this activity, i.e., by attempting to enhance alpha activity and reduce delta activity.

**Where Can I Get More Information?**

MedlinePlus has a Web page on tinnitus: http://www.nlm.nih.gov/medlineplus/tinnitus.html

The Welcome Gateways has a selection of Web sites on tinnitus: http://omni.ac.uk/browse/mesh/D014012.html

The American Tinnitus Association also offers information on tinnitus: http://www.ata.org

## Abbreviations

MEG: magnetoencephalography
